# Tsallis Entropy of Product MV-Algebra Dynamical Systems

**DOI:** 10.3390/e20080589

**Published:** 2018-08-09

**Authors:** Dagmar Markechová, Beloslav Riečan

**Affiliations:** 1 Department of Mathematics, Faculty of Natural Sciences, Constantine the Philosopher University in Nitra, A. Hlinku 1, SK-949 01 Nitra, Slovakia; 2 Department of Mathematics, Faculty of Natural Sciences, Matej Bel University, Tajovského 40, SK-974 01 Banská Bystrica, Slovakia; 3 Mathematical Institute, Slovak Academy of Sciences, Štefánikova 49, SK-814 73 Bratislava, Slovakia

**Keywords:** product MV-algebra, partition, Tsallis entropy, conditional Tsallis entropy, dynamical system

## Abstract

This paper is concerned with the mathematical modelling of Tsallis entropy in product MV-algebra dynamical systems. We define the Tsallis entropy of order α, where α∈(0,1)∪(1,∞), of a partition in a product MV-algebra and its conditional version and we examine their properties. Among other, it is shown that the Tsallis entropy of order α, where α>1, has the property of sub-additivity. This property allows us to define, for α>1, the Tsallis entropy of a product MV-algebra dynamical system. It is proven that the proposed entropy measure is invariant under isomorphism of product MV-algebra dynamical systems.

## 1. Introduction

The Shannon entropy [1] is the foundational concept of information theory (cf. [2]). We remind readers that if an experiment has *k* outcomes with probabilities $p_{1},p_{2},\ldots ,p_{k},$ then its Shannon entropy is defined as the sum $\sum_{i=1}^{k}s(p_{i}),$ where s:[0,1]→[0,∞) is Shannon’s entropy function defined by:

(1) s(x)=−xlogx,

for every x∈[0,1] (0log0 is defined to be 0). Many years later, the Shannon entropy was exploited surprisingly in a completely different field, namely, in dynamical systems. Recall that by a dynamical system in the sense of classical probability theory, we understand a system (Ω,Σ,μ,T), where (Ω,Σ,μ) is a probability space, and T:Ω→Ω is a measure μ preserving transformation. The entropy of dynamical systems was introduced by Kolmogorov and Sinai [3,4] as an invariant for distinguishing them. Namely, if two dynamical systems are isomorphic, then they have the same entropy. In this way Kolmogorov and Sinai showed the existence of non-isomorphic Bernoulli shifts.

The successful using the Kolmogorov-Sinai entropy of dynamical systems has led to an intensive study of alternative entropy measures of dynamical systems. We note that in Reference [5], the concept of logical entropy $H_{l}(T)$ of a dynamical system (Ω,Σ,μ,T) was introduced and studied. It has been shown that by replacing Shannon’s entropy function by the function l:[0,1]→[0,∞) defined by:

(2) $$l(x)=x-x^{2},$$

for every x∈[0,1], we obtain the results analogous to the case of Kolmogorov-Sinai entropy theory. The logical entropy $H_{l}(T)$ is invariant under isomorphism of dynamical systems; so it can be used as an alternative instrument for distinguishing them. For some other recently published results regarding the logical entropy measure, we refer, for example, to References [6,7,8,9,10,11,12,13,14,15].

In fact, all of the above mentioned studies are possible in the Kolmogorov probability theory based on the modern integration theory. This allows us to describe and study some of the problems associated with uncertainty. In Reference [16], Zadeh presented another approach to uncertainty when he introduced the concept of a fuzzy set. Whereas the Kolmogorov probability applications are based on objective measurements, the Zadeh fuzzy set theory is based on subjective improvements. One of the first Zadeh papers on the fuzzy set theory was devoted to probability of fuzzy sets (cf. [17]), and therefore, the entropy of fuzzy dynamical systems has also been studied (cf. [18,19,20,21]). We recall that the fuzzy set is a mapping f:Ω→[0,1], hence, the fuzzy partition of Ω is a family of fuzzy sets $A=\left\{f_{1},f_{2},\ldots ,f_{k}\right\}$ such that $\sum_{i=1}^{k}f_{i}=1.$ Again we can meet the Shannon formula: $H(A)=-\sum_{i=1}^{k}p_{i}\log p_{i},$ where $p_{i}=\int_{\Omega }f_{i}d\mu$ (cf. [21]).

Anyway the most useful tool for describing multivalued processes is an MV-algebra [22], especially after its Mundici’s characterization as an interval in a lattice ordered group (cf. [23,24]). At present, this structure is being investigated by many researchers, and it is natural that there are results also regarding the entropy in this structure; we refer, for instance, to References [25,26]. A probability theory was also investigated on MV-algebras; for a review see Reference [27]. Of course, in some probability problems it is needed to introduce a product on an MV-algebra, an operation outside the corresponding group addition. The operation of a product on an MV-algebra was introduced independently by Riečan [28] from the point of view of probability and Montagna [29] from the point of view of mathematical logic. We note that the notion of product MV-algebra generalizes some families of fuzzy sets; an example of product MV-algebra is a full tribe of fuzzy sets (see e.g., [30]).

The appropriate entropy theory of Shannon and Kolmogorov-Sinai type for the product MV-algebras was created in References [31,32]. The logical entropy, the logical divergence and the logical mutual information of partitions in a product MV-algebra were studied in Reference [8]. In the present paper, we extend the study of entropy in product MV-algebras to the case of Tsallis entropy.

The concept of Tsallis entropy was introduced in 1988 by Constantino Tsallis [33] as a base for generalizing the usual statistical mechanics. In its form it is identical with the Havrda-Charvát structural α—entropy [34], introduced in 1967 in the framework of information theory. If $P=\left\{p_{1},p_{2},\ldots ,p_{k}\right\}$ is a probability distribution, then its Tsallis entropy of order α, where α∈(0,1)∪(1,∞), is defined as the number:

(3) $$T_{\alpha }(P)=\frac{1}{\alpha -1}\left(1-\sum_{i=1}^{k}p_{i}{}^{\alpha }\right).$$

The entropic index α describes the deviation of Tsallis entropy from the standard Shannon one. Evidently, if we define, for α∈(0,1)∪(1,∞), the function $l_{\alpha }:[0,1]\to [0,\infty )$ by:

(4) $$l_{\alpha }(x)=\frac{1}{\alpha -1}\left(x-x^{\alpha }\right),$$

for every x∈[0,1], then the Formula (3) can be written in the following form:

(5) $$T_{\alpha }(P)=\sum_{i=1}^{k}l_{\alpha }(p_{i}).$$

Putting α=2 in Equation (5), we obtain:

$$T_{2}(P)=\sum_{i=1}^{k}l_{2}(p_{i})=\sum_{i=1}^{k}(p_{i}-p_{i}{}^{2})=1-\sum_{i=1}^{k}p_{i}{}^{2},$$

which is the logical entropy of a probability distribution $P=\left\{p_{1},p_{2},\ldots ,p_{k}\right\}$ defined and studied in Reference [6].

The Tsallis entropy is the most important quantity among Tsallis’ statistics, which form the foundation of nonextensive statistical mechanics of complex systems; for more details, see Reference [35]. The Tsallis statistics are used to describe systems exhibiting long-range correlations, memory, or fractal properties; their applications have been found for a wide range of phenomena in diverse disciplines such as physics, geophysics, chemistry, biology, economics, medicine, etc. [36,37,38,39,40,41,42,43,44,45,46,47,48]. They are also applicable to large domains in communication systems (cf. [49]).

In this article we continue studying entropy in a product MV-algebra, by defining and studying the Tsallis entropy of a partition in a product MV-algebra and the Tsallis entropy of product MV-algebra dynamical systems. The rest of the paper is structured as follows. In the following section, preliminaries and related works are given. Our main results are discussed in Section 3, Section 4 and Section 5. In Section 3, we define and study the Tsallis entropy of a partition in a product MV-algebra. In Section 4, we introduce the concept of conditional Tsallis entropy of partitions in a product MV-algebra and examine its properties. It is shown that the proposed definitions of Tsallis entropy are consistent, in the case of the limit of α going to 1, with the Shannon entropy of partitions studied in Reference [31]. Section 5 is devoted to the study of Tsallis entropy of product MV-algebra dynamical systems. It is proven that the suggested entropy measure is invariant under isomorphism of product MV-algebra dynamical systems. The last section contains a brief summary.

## 2. Preliminaries 

We begin with recalling the definitions of the basic notions and some known results used in the paper. For defining the notion of MV-algebra, various (but of course equivalent) axiom systems were used (see e.g., [28,50,51]). In this paper, the definition of MV-algebra given by Riečan in Reference [52] is used, which is based on the Mundici representation theorem [23,24]. In view of the Mundici theorem, any MV-algebra may be considered to be an interval of an abelian lattice ordered group. Recall that by an abelian lattice ordered group [53], we mean a triplet (L,+,≤), where (L,+) is an abelian group, (L,≤) is a partially ordered set being a lattice and, for every x,y,z∈L, x≤y ⟹ x+z≤y+z.

**Definition** **1**  ([52])**.** *An MV-algebra is an algebraic structure A=(A,⊕,∗,0,u) satisfying the following conditions:*

*(i)*  *there exists an abelian lattice ordered group (L,+,≤) such that A=[0,u]={x∈L;0≤x≤u}, where* 0 *is the neutral element of (L,+) and u is a strong unit of L (i.e., u∈L such that u>0 and to each x∈L there exists a natural number n with the property x≤nu);*
*(ii)*  ⊕ and ∗ are binary operations on A satisfying the following identities: x⊕y=(x+y)∧u,x∗y=(x+y−u)∨0.

**Definition** **2**  ([27])**.** *A state on an MV-algebra A=(A,⊕,∗,0,u) is a mapping s:A→[0,1] with the following two properties:*

*(i)*  s(u)=1;
*(ii)*  *if*x,y∈A*such that*x+y≤u,*then*s(x+y)=s(x)+s(y).

**Definition** **3**  ([28])**.** *A product MV-algebra is an algebraic structure* (A,⊕,∗,⋅,0,u), *where* (A,⊕,∗,0,u) *is an MV-algebra and* ⋅ *is an associative and abelian binary operation on A with the following properties:*

*(i)*  *for every*x∈A,u⋅x=x;
*(ii)*  *if*x,y,z∈A*such that*x+y≤u,*then*z⋅x+z⋅y≤u,*and*z⋅(x+y)=z⋅x+z⋅y.

In the following text, we will briefly write (A,⋅) instead of (A,⊕,∗,⋅,0,u). A relevant probability theory for the product MV-algebras was developed by Riečan in Reference [54]; the entropy theory of Shannon and Kolmogorov-Sinai type for the product MV-algebras was proposed in References [31,32]. The logical entropy theory for the product MV-algebras was proposed in Reference [8]. We present the main idea and some results of these theories that will be used in the following text.

By a partition in a product MV-algebra (A,⋅), we mean a *k*-tuple $X=(x_{1},x_{2},\ldots ,x_{k})$ of (not necessarily different) members of *A* satisfying the condition $x_{1}+x_{2}+\ldots +x_{k}=u.$ Let $X=(x_{1},x_{2},\ldots ,x_{k}),$ and $Y=(y_{1},y_{2},\ldots ,y_{l})$ be two partitions in (A,⋅). We say that Y is a refinement of X, and we write X≺Y, if there exists a partition {β(1),β(2),…,β(k)} of the set {1,2,…,l} such that $x_{i}=\sum_{j\in \beta (i)}y_{j},$ for i=1,2,…,k. Further, we define the join X∨Y of X and Y as an *r*-tuple (where r=k⋅l) consisting of the members $x_{ij}=x_{i}\cdot y_{j},$ i=1,2,…,k, j=1,2,…,l. Since $\sum_{i=1}^{k}\sum_{j=1}^{l}x_{i}\cdot y_{j}=$ $\left(\sum_{i=1}^{k}x_{i}\right)\cdot \left(\sum_{j=1}^{l}y_{j}\right)=u\cdot u=u,$ the *r*-tuple X∨Y is a partition in (A,⋅). It represents an experiment consisting of the realization of X and Y.

**Example** **1.** *Consider a probability space* (Ω,Σ,μ) *and define* $A=\left\{I_{E};E\in \Sigma \right\},$ *where* $I_{E}:\Omega \to \left\{0,1\right\}$ *stands for the indicator function of the set* E∈Σ. *The family A is closed under the product of indicator functions and it is a special case of product MV-algebras. The map* s:A→[0,1] *defined, for every* $I_{E}$ *of* A, *by* $s(I_{E})=\mu (E),$ *is a state on the considered product MV-algebra* (A,⋅). *Evidently, if* $\left\{E_{1},E_{2},\ldots ,E_{k}\right\}$ *is a measurable partition of* (Ω,Σ,μ), *then the k-tuple* $\left(I_{E_{1}},I_{E_{2}},\ldots ,I_{E_{k}}\right)$ *is a partition in the product MV-algebra* (A,⋅).

**Example** **2.** *Consider a probability space* (Ω,Σ,μ) *and the family A of all* Σ *-measurable functions* f:Ω→[0,1], *so called full tribe of fuzzy sets (cf., e.g., [21,30]). The family A is closed under the natural product of fuzzy sets and it is an important case of product MV-algebras. The mapping* s:A→[0,1] *defined, for every* f∈A, *by the formula* $s(f)=\int_{\Omega }fd\mu ,$ *is a state on the product MV-algebra* (A,⋅). *The concept of a partition in the product MV-algebra* (A,⋅) *Tcoincides with the notion of a fuzzy partition (cf. [21]).*

The definition of entropy of Shannon type of a partition in a product MV-algebra was introduced in [31] as follows.

**Definition** **4.** *Let* $X=(x_{1},x_{2},\ldots ,x_{k})$ *be any partition in a product MV-algebra* (A,⋅) *and* s:A→[0,1] *be a state. Then the entropy of* X *with respect to* s *is defined by:*

(6) $$H_{s}(X)=-\sum_{i=1}^{k}s(x_{i})\cdot \log s(x_{i}).$$

*If* $X=(x_{1},x_{2},\ldots ,x_{k}),$ *and* $Y=(y_{1},y_{2},\ldots ,y_{l})$ *are two partitions in* (A,⋅), *then the conditional entropy of* X *given* $y_{j}\in Y$ *is defined by:*

$$H_{s}(X/y_{j})=-\sum_{i=1}^{k}s(x_{i}/y_{j})\cdot \log s(x_{i}/y_{j}),$$

*where:*

(7) $$s(x_{i}/y_{j})=\left\{ \begin{array}{lll} \frac{s(x_{i}\cdot y_{j})}{s(y_{j})}, & \mathrm{if} & s(y_{j})>0;\\ 0, & \mathrm{if} & s(y_{j})=0. \end{array}\right.$$

The conditional entropy of X given Y is defined by:

(8) $$H_{s}(X/Y)=\sum_{j=1}^{l}s(y_{j})\cdot H_{s}(X/y_{j})=-\sum_{i=1}^{k}\sum_{j=1}^{l}s(x_{i}\cdot y_{j})\cdot \log\frac{s(x_{i}\cdot y_{j})}{s(y_{j})}.$$

It is used the standard convention that $0\log\frac{0}{x}=0$ if x≥0. The basis of the logarithm may be any positive real number, but as a rule logarithms to the basis 2 are taken; the entropy is then expressed in bits. If the natural logarithms are taken in the definition, then the entropy is expressed in nats. The entropy of partitions in a product MV-algebra possesses properties corresponding to properties of Shannon’s entropy of measurable partitions; more details can be found in Reference [31].

The definition of logical entropy of a partition in a product MV-algebra was introduced in Reference [8] as follows.

**Definition** **5.** *Let* $X=(x_{1},x_{2},\ldots ,x_{k})$ *be a partition in a product MV-algebra* (A,⋅), *and* s:A→[0,1] *be a state. Then the logical entropy of* X *with respect to* s *is defined by:*

(9) $$H_{{}^{l}}^{s}(X)=\sum_{i=1}^{k}l(s(x_{i})),$$

*where* l:[0,1]→[0,∞) *is the logical entropy function defined by Equation (2). If* $X=(x_{1},x_{2},\ldots ,x_{k}),$ *and* $Y=(y_{1},y_{2},\ldots ,y_{l})$ *are two partitions in* (A,⋅), *then the conditional logical entropy of* X *given* Y *is defined by:*

(10) $$H_{{}^{l}}^{s}(X/Y)=\sum_{j=1}^{l}s{\left(y_{j}\right)}^{2}-\sum_{i=1}^{k}\sum_{j=1}^{l}s{\left(x_{i}\cdot y_{j}\right)}^{2}.$$

## 3. The Tsallis Entropy of Partitions in a Product MV-Algebra

We begin this section with the definition of Tsallis entropy of a partition in a product MV-algebra (A,⋅), and then we will examine its properties. In the following, we will suppose that s:A→[0,1] is a state.

**Definition** **6.** *Let*$X=(x_{1},x_{2},\ldots ,x_{k})$*be a partition in a product MV-algebra*(A,⋅).*Then we define the Tsallis entropy of order*α,*where*α∈(0,1)∪(1,∞),*of the partition*X*with respect to*s by:

(11) $$T_{\alpha }^{s}(X)=\frac{1}{\alpha -1}\left(1-\sum_{i=1}^{k}s{\left(x_{i}\right)}^{\alpha }\right).$$

**Remark** **1.** *Let us consider the function* $l_{\alpha }:[0,1]\to [0,\infty )$ *defined by Equation (4). Since* $\sum_{i=1}^{k}s(x_{i})=1,$ *the formula (11) can be expressed in the following form:*

(12) $$T_{\alpha }^{s}(X)=\sum_{i=1}^{k}l_{\alpha }\left(s(x_{i})\right).$$

*Evidently, if we put* α=2, *the logical entropy* $H_{{}^{l}}^{s}(X)$ *is obtained. It is possible to verify that the function* $l_{\alpha }$ *is, for every* α∈(0,1)∪(1,∞), *a non-negative function. Namely, if* α∈(0,1), *then we have* $x^{\alpha }\geq x,$ *for every* x∈[0,1], *hence* $l_{\alpha }(x)=\frac{1}{\alpha -1}\left(x-x^{\alpha }\right)\geq 0,$ *for every* x∈[0,1]. *On the other hand, for* α>1, *we have* $x^{\alpha }\leq x,$ *for every* x∈[0,1], *hence* $l_{\alpha }(x)=\frac{1}{\alpha -1}\left(x-x^{\alpha }\right)\geq 0,$ *for every* x∈[0,1].

**Example** **3.** *Let* (A,⋅) *be any product MV-algebra. Let us consider the partition* E=(u) *representing an experiment resulting in a certain event. Then* E≺X, *for every partition X in* (A,⋅), *and* $T_{\alpha }^{s}(E)=l_{\alpha }\left(s(u)\right)=l_{\alpha }(1)=0.$

**Theorem** **1.** *Let* $X=(x_{1},x_{2},\ldots ,x_{k})$ *be any partition in a product MV-algebra* (A,⋅). *Then:*

$$0\leq T_{\alpha }^{s}(X)\leq \frac{1}{\alpha -1}\left(1-k^{1-\alpha }\right).$$

*The equality* $T_{\alpha }^{s}(X)=\frac{1}{\alpha -1}\left(1-k^{1-\alpha }\right)$ *holds if and only if the state s is uniform over X, i.e., if and only if* $s(x_{i})=\frac{1}{k},$ *for* i=1,2,…,k.

**Proof.**  The inequality $T_{\alpha }^{s}(X)\geq 0$ follows from the non-negativity of function $l_{\alpha },$ so it is sufficient to prove the second assertion. We will use the Jensen inequality. It is easy to verify that the function $l_{\alpha }$ is concave, therefore, applying the Jensen inequality, we have:

$$l_{\alpha }\left(\frac{1}{k}\sum_{i=1}^{k}s(x_{i})\right)\geq \sum_{i=1}^{k}\frac{1}{k}l_{\alpha }\left(s(x_{i})\right)$$

with the equality if and only if $s(x_{1})=s(x_{2})=\ldots =s(x_{k}).$ Since $\sum_{i=1}^{k}s(x_{i})=1,$ it follows that:

$$T_{\alpha }^{s}(X)=\sum_{i=1}^{k}l_{\alpha }\left(s(x_{i})\right)\leq k\cdot l_{\alpha }\left(\frac{1}{k}\sum_{i=1}^{k}s(x_{i})\right)=k\cdot l_{\alpha }\left(\frac{1}{k}\right)=\frac{k}{\alpha -1}\left(\frac{1}{k}-{\left(\frac{1}{k}\right)}^{\alpha }\right)=\frac{1}{\alpha -1}\left(1-k^{1-\alpha }\right).$$

The equality holds if and only if $s(x_{1})=s(x_{2})=\ldots =s(x_{k}),$ i.e., if and only if $s(x_{i})=\frac{1}{k},$ for i=1,2,…,k. ☐

The following propositions will be needed for the proofs of our results.

**Proposition** **1.** *Let* X,Y,Z *be partitions in a product MV-algebra* (A,⋅). *Then:*

*(i)*  X≺X∨Y;
*(ii)*  X≺Y *implies* X∨Z≺Y∨Z.

**Proof.**  For the proof, see Reference [8]. ☐

**Proposition** **2.** *Let* X,Y,V,Z *be partitions in a product MV-algebra* (A,⋅) *such that* X≺Y, *and* V≺Z. *Then* X∨V≺Y∨Z.

**Proof.**  Let $X=(x_{1},x_{2},\ldots ,x_{k}),$ $Y=(y_{1},y_{2},\ldots ,y_{l}),$ $V=(v_{1},v_{2},\ldots ,v_{m}),$ $Z=(z_{1},z_{2},\ldots ,z_{n}),$ X≺Y, V≺Z. Then there exists a partition {β(1),β(2),…,β(k)} of the set {1,2,…,l} such that $x_{i}=\sum_{j\in \beta \left(i\right)}y_{j},$ for i=1,2,…,k, and there exists a partition {γ(1),γ(2),…,γ(m)} of the set {1,2,…,n} such that $v_{r}=\sum_{k\in \gamma \left(r\right)}z_{k},$ for r=1,2,…,m. Put δ(i,r)={(j,k);j∈β(i),k∈γ(r)}, for i=1,2,…,k, r=1,2,…,m. We get:

$$x_{i}\cdot v_{r}=\left(\sum_{j\in \beta \left(i\right)}y_{j}\right)\cdot \left(\sum_{k\in \gamma \left(r\right)}z_{k}\right)=\sum_{(j,k)\in \delta \left(i,r\right)}y_{j}\cdot z_{k},$$

for i=1,2,…,k, r=1,2,…,m, what means that X∨V≺Y∨Z. ☐

**Proposition** **3.** *Let* $X=(x_{1},x_{2},\ldots ,x_{k})$ *be a partition in a product MV-algebra* (A,⋅), *and* s:A→[0,1] *be a state. Then:*

*(i)*  $\sum_{i=1}^{k}s(x_{i}\cdot y)=s(y),$ *for every* y∈A;
*(ii)*  $\sum_{i=1}^{k}s(x_{i}/y)=1,$ *for every* y∈A *with* s(y)>0.

**Proof.**  For the proof of the claim (i), see [8]. If y∈A with s(y)>0, then using the previous equality, we obtain:

$$\sum_{i=1}^{k}s(x_{i}/y)=\frac{1}{s(y)}\sum_{i=1}^{k}s(x_{i}\cdot y)=\frac{s(y)}{s(y)}=1.\;☐$$

**Theorem** **2.** *Let* X,Y *be partitions in a product MV-algebra* (A,⋅) *such that* X≺Y. *Then* $T_{\alpha }^{s}(X)\leq T_{\alpha }^{s}(Y).$

**Proof.**  Suppose that $X=(x_{1},x_{2},\ldots ,x_{k}),$ $Y=(y_{1},y_{2},\ldots ,y_{l}),$ X≺Y. Then there exists a partition {β(1),β(2),…,β(k)} of the set {1,2,…,l} such that $x_{i}=\sum_{j\in \beta \left(i\right)}y_{j},$ for i=1,2,…,k. Hence $s(x_{i})=$ $s\left(\sum_{j\in \beta (i)}y_{j}\right)=\sum_{j\in \beta (i)}s(y_{j}),$ for i=1,2,…,k. Consider the case when α∈(1,∞). Then:

$$s{\left(x_{i}\right)}^{\alpha }={\left(\sum_{j\in \beta (i)}s(y_{j})\right)}^{\alpha }\geq \sum_{j\in \beta (i)}s(y_{j}{)}^{\alpha },$$

for i=1,2,…,k. Summing both sides of the above inequality over *i*, we get:

$$\sum_{i=1}^{k}s{\left(x_{i}\right)}^{\alpha }\geq \sum_{i=1}^{k}\sum_{j\in \beta (i)}s(y_{j}{)}^{\alpha }=\sum_{j=1}^{l}s{\left(y_{j}\right)}^{\alpha }.$$

In this case we have $\frac{1}{\alpha -1}>0,$ hence:

$$T_{\alpha }^{s}(X)=\frac{1}{\alpha -1}\left(1-\sum_{i=1}^{k}s{\left(x_{i}\right)}^{\alpha }\right)\leq \frac{1}{\alpha -1}\left(1-\sum_{j=1}^{l}s{\left(y_{j}\right)}^{\alpha }\right)=T_{\alpha }^{s}(Y).$$

The case of α∈(0,1) can be obtained in the same way. ☐

As an immediate consequence of the previous theorem and Proposition 1, we obtain the following result.

**Corollary** **1.** *For every partitions* X,Y *in a product MV-algebra* (A,⋅), *it holds:*

$$T_{\alpha }^{s}(X\vee Y)\geq \max\left[T_{\alpha }^{s}(X),T_{\alpha }^{s}(Y)\right].$$

**Proposition** **4.** *Let* $X=(x_{1},x_{2},\ldots ,x_{k}),$ *and* $Y=(y_{1},y_{2},\ldots ,y_{l})$ *be partitions in a product MV-algebra* (A,⋅). *Then, for* α>1, *it holds:*

$$\sum_{j=1}^{l}s{\left(y_{j}\right)}^{\alpha }\sum_{i=1}^{k}l_{\alpha }\left(s(x_{i}/y_{j})\right)\leq T_{\alpha }^{s}(X).$$

**Proof.**  Applying the Jensen inequality, we have:

$$\sum_{j=1}^{l}s(y_{j})\cdot l_{\alpha }\left(s(x_{i}/y_{j})\right)\leq l_{\alpha }\left(\sum_{j=1}^{l}s(y_{j})\cdot s(x_{i}/y_{j})\right)=l_{\alpha }\left(\sum_{j=1}^{l}s(x_{i}\cdot y_{j})\right)=l_{\alpha }\left(s(x_{i})\right),$$

for i=1,2,…,k, and consequently:

(13) $$\sum_{j=1}^{l}s(y_{j})\cdot \sum_{i=1}^{k}l_{\alpha }\left(s(x_{i}/y_{j})\right)\leq \sum_{i=1}^{k}l_{\alpha }\left(s(x_{i})\right)=T_{\alpha }^{s}(X).$$

The assumption that α>1 implies the inequality $s{\left(y_{j}\right)}^{\alpha }\leq s(y_{j}),$ for j=1,2,…,l. The function $l_{\alpha }$ is non-negative, therefore, for j=1,2,…,l, we get:

$$s{\left(y_{j}\right)}^{\alpha }\sum_{i=1}^{k}l_{\alpha }\left(s(x_{i}/y_{j})\right)\leq s(y_{j})\sum_{i=1}^{k}l_{\alpha }\left(s(x_{i}/y_{j})\right),$$

and consequently:

$$\sum_{j=1}^{l}s{\left(y_{j}\right)}^{\alpha }\sum_{i=1}^{k}l_{\alpha }\left(s(x_{i}/y_{j})\right)\leq \sum_{j=1}^{l}s(y_{j})\sum_{i=1}^{k}l_{\alpha }\left(s(x_{i}/y_{j})\right).$$

The last inequality combined with (13) yields the claim. ☐

**Theorem** **3.** *Let* X,Y *be partitions in a product MV-algebra* (A,⋅). *Then, for* α>1, *it holds:*

$$T_{\alpha }^{s}(X\vee Y)\leq T_{\alpha }^{s}(X)+T_{\alpha }^{s}(Y).$$

**Proof.**  Suppose that $X=(x_{1},x_{2},\ldots ,x_{k}),$ $Y=(y_{1},y_{2},\ldots ,y_{l}).$ Let us calculate:

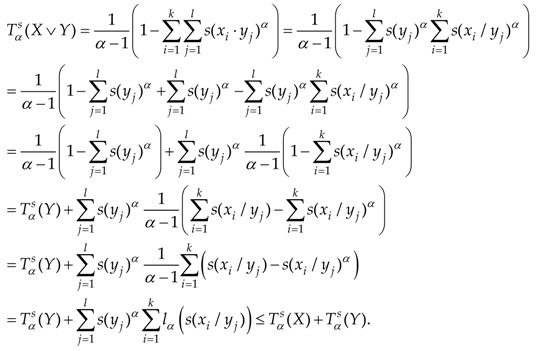
 In the last step we used Proposition 4. ☐

**Example** **4.** *Let us consider the family A of all Borel measurable functions* f:[0,1]→[0,1], *and define in A the operation* ⋅ *as the natural product of fuzzy sets. Then the system* (A,⋅) *is a product MV-algebra. In addition, we define a state* s:A→[0,1] *by the formula* $s(f)=\int_{0}^{1}f(x)dx,$ *for every* f∈A, *and consider the pairs* $X=(f_{1},f_{2}),$ $Y=(g_{1},g_{2}),$ *where* $f_{1}(x)=x,$ $f_{2}(x)=1-x,$ $g_{1}(x)=x^{2},$ $g_{2}(x)=1-x^{2},$ *for every* x∈[0,1]. *Evidently, X and Y are partitions in the product MV-algebra* (A,⋅). *By elementary calculations we get that they have the state values* $\frac{1}{2},\frac{1}{2}$ *and* $\frac{1}{3},\frac{2}{3}$ *of the corresponding elements, respectively. The partition* $X\vee Y=(f_{1}\cdot g_{1},f_{1}\cdot g_{2},f_{2}\cdot g_{1},f_{2}\cdot g_{2})$ *has the state values* $\frac{1}{4},\frac{1}{4},\frac{1}{12},\frac{5}{12}$ *of the corresponding elements. We want to find out whether the statement of the previous theorem is true in the case under consideration. Using the formula (11), it can be computed that* $T_{2}^{s}(X)=0.5,$ $T_{2}^{s}(Y)\dot{=}0.4444,$ $T_{2}^{s}(X\vee Y)\dot{=}0.6944,$ $T_{3}^{s}(X)=0.375,$ $T_{3}^{s}(Y)\dot{=}0.3333,$ $T_{3}^{s}(X\vee Y)\dot{=}0.4479.$ *It holds* $T_{2}^{s}(X\vee Y)<T_{2}^{s}(X)+T_{2}^{s}(Y),$ *and* $T_{3}^{s}(X\vee Y)<T_{3}^{s}(X)+T_{3}^{s}(Y),$ *which is consistent with the assertion of Theorem 3. Put* $\alpha =\frac{1}{2}.$ *We obtain:* $T_{1/2}^{s}(X)\dot{=}0.8284,$ $T_{1/2}^{s}(Y)\dot{=}0.7877,$ $T_{1/2}^{s}(X\vee Y)\dot{=}1.8683.$ *It can be seen that* $T_{1/2}^{s}(X\vee Y)>$ $T_{1/2}^{s}(X)+T_{1/2}^{s}(Y).$ *The result means that the Tsallis entropy* $T_{\alpha }^{s}(X)$ *of order* α∈(0,1) *does not have the property of sub-additivity.*

One of the most important properties of Shannon entropy is additivity. In the following theorem it is shown that the Tsallis entropy $T_{\alpha }^{s}(X)$ does not have the property of additivity; it satisfies the following weaker property of pseudo-additivity.

**Theorem** **4.** *If partitions* X,Y *in a product MV-algebra* (A,⋅) *are statistically independent with respect to* s, *i.e.,* s(x⋅y)=s(x)⋅s(y), *for every* x∈X, *and* y∈Y, *then:*

$$T_{\alpha }^{s}(X\vee Y)=T_{\alpha }^{s}(X)+T_{\alpha }^{s}(Y)+(1-\alpha )\cdot T_{\alpha }^{s}(X)\cdot T_{\alpha }^{s}(Y).$$

**Proof.**  Suppose that $X=(x_{1},x_{2},\ldots ,x_{k}),$ $Y=(y_{1},y_{2},\ldots ,y_{l}).$ Let us calculate:

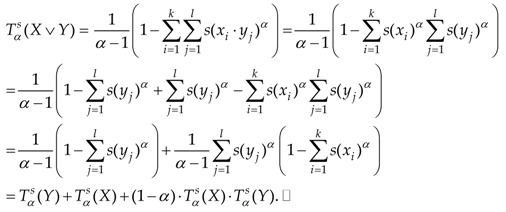

In the last part of this section, we will prove the concavity of Tsallis entropy $T_{\alpha }^{s}(X)$ on the family of all states defined on a given product MV-algebra (A,⋅).

**Proposition** **5.** *Let* $s_{1},s_{2}$ *be two states defined on a common product MV-algebra* (A,⋅). *Then, for every real number* λ∈[0,1], *the map* $\lambda s_{1}+(1-\lambda )s_{2}:A\to [0,1]$ *is a state on* (A,⋅).

**Proof.**  The proof is simple, so it is omitted. ☐

**Theorem** **5.** *Let* $s_{1},s_{2}$ *be two states defined on a common product MV-algebra* (A,⋅). *Then, for every partition* X *in a product MV-algebra* (A,⋅), *and for every real number* λ∈[0,1], *the following inequality holds:*

$$\lambda T_{\alpha }^{s_{1}}(X)+(1-\lambda )T_{\alpha }^{s_{2}}(X)\leq T_{\alpha }^{\lambda s_{1}+(1-\lambda )s_{2}}(X).$$

**Proof.**  Assume that $X=(x_{1},x_{2},\ldots ,x_{k}).$ The function $l_{\alpha }$ is concave, therefore, for every real number λ∈[0,1], we get:

$$\begin{array}{l} \lambda T_{\alpha }^{s_{1}}(X)+(1-\lambda )T_{\alpha }^{s_{2}}(X)=\lambda \sum_{i=1}^{k}l_{\alpha }\left(s_{1}(x_{i})\right)+(1-\lambda )\sum_{i=1}^{k}l_{\alpha }\left(s_{2}(x_{i})\right)\\ =\sum_{i=1}^{k}\left(\lambda l_{\alpha }\left(s_{1}(x_{i})\right)+(1-\lambda )l_{\alpha }\left(s_{2}(x_{i})\right)\right)\leq \sum_{i=1}^{k}l_{\alpha }\left(\lambda s_{1}(x_{i})+(1-\lambda )s_{2}(x_{i})\right)\\ =\sum_{i=1}^{k}l_{\alpha }\left(\left(\lambda s_{1}+(1-\lambda )s_{2})(x_{i}\right)\right)=T_{\alpha }^{\lambda s_{1}+(1-\lambda )s_{2}}(X).\;☐ \end{array}$$

As a consequence of Theorem 5, we get the concavity of the logical entropy $H_{{}^{l}}^{s}(X)$ as a function of *s*. The result of the previous theorem is illustrated in the following example.

**Example** **5.** *Consider the product MV-algebra* (A,⋅) *from Example 4 and the real functions* $G_{1},G_{2}$ *defined by the equalities* $G_{1}(x)=x,G_{2}(x)=x^{2},$ *for every real number* x. *We define two states* $s_{1}:A\to [0,1],$ $s_{2}:A\to [0,1]$ *by the formulas* $s_{1}(f)=\int_{0}^{1}f(x)dG_{1}(x)=\int_{0}^{1}f(x)dx,$ $s_{2}(f)=\int_{0}^{1}f(x)dG_{2}(x)=\int_{0}^{1}f(x)2xdx,$ *for every* f *of* A. *Further, we consider the partition* $X=\left(I_{\left[0,\frac{1}{3}\right)},I_{\left[\frac{1}{3},1\right]}\right)$ *in* (A,⋅). *By simple calculation we get that it has the* $s_{1}$ *-state values* $\frac{1}{3},\frac{2}{3}$ *of the corresponding elements, and the* $s_{2}$ *-state values* $\frac{1}{9},\frac{8}{9}$ *of the corresponding elements. In the previous theorem we put* λ=0.2. *We will show that, for the chosen* α∈(0,1)∪(1,∞), *the following inequality holds:*

(14) $$0.2\cdot T_{\alpha }^{s_{1}}(X)+0.8\cdot T_{\alpha }^{s_{2}}(X)\leq T_{\alpha }^{0.2s_{1}+0.8s_{2}}(X).$$

*Put* $\alpha =\frac{1}{2}.$ *We calculated that* $T_{1/2}^{s_{1}}(X)\dot{=}0.7877,$ $T_{1/2}^{s_{2}}(X)\dot{=}0.5523,$ *and* $T_{1/2}^{0.2s_{1}+0.8s_{2}}(X)\dot{=}0.6267.$ *One can easily check that in this case:*

$$0.2\cdot T_{1/2}^{s_{1}}(X)+0.8\cdot T_{1/2}^{s_{2}}(X)<T_{1/2}^{0.2s_{1}+0.8s_{2}}(X).$$

*For the case of* α=2, *i.e., for the logical entropy, we get:* $T_{2}^{s_{1}}(X)\dot{=}0.4444,$ $T_{2}^{s_{2}}(X)\dot{=}0.1975,$ $T_{2}^{0.2s_{1}+0.8s_{2}}(X)\dot{=}0.2627,$ *and for the case of* α=3, *we obtain:* $T_{3}^{s_{1}}(X)\dot{=}0.3333,$ $T_{3}^{s_{2}}(X)\dot{=}0.148148,$ $T_{3}^{0.2s_{1}+0.8s_{2}}(X)\dot{=}0.19704.$ *One can easily check that in both cases the inequality (14) holds.*

## 4. The Conditional Tsallis Entropy of Partitions in a Product MV-Algebra

In this section we introduce and study the concept of conditional Tsallis entropy of partitions in a product MV-algebra (A,⋅).

**Definition** **7.**  *Let* $X=(x_{1},x_{2},\ldots ,x_{k}),$ *and* $Y=(y_{1},y_{2},\ldots ,y_{l})$ *be partitions in a product MV-algebra* (A,⋅). *We define the conditional Tsallis entropy of order* α, *where* α∈(0,1)∪(1,∞), *of X given Y as the number:*

(15) $$T_{\alpha }^{s}(X/Y)=\frac{1}{\alpha -1}\left(\sum_{j=1}^{l}s{\left(y_{j}\right)}^{\alpha }-\sum_{i=1}^{k}\sum_{j=1}^{l}s{\left(x_{i}\cdot y_{j}\right)}^{\alpha }\right).$$

**Remark** **2.** *Evidently, if we put α=2, then we obtain the conditional logical entropy of X given Y defined by Equation (10).*

At α=1 the value of $T_{\alpha }^{s}(X/Y)$ is undefined because it gives the shape $\frac{0}{0}.$ In the following theorem it is shown that for α→1 the conditional Tsallis entropy $T_{\alpha }^{s}(X/Y)$ tends to the conditional Shannon entropy $H_{s}(X/Y)$ defined by the formula (8), when the natural logarithm is taken in this formula.

**Theorem** **6.** *Let* $X=(x_{1},x_{2},\ldots ,x_{k}),$ *and* $Y=(y_{1},y_{2},\ldots ,y_{l})$ *be partitions in a product MV-algebra* (A,⋅). *Then:*

$$\underset{\alpha \to 1}{\lim}T_{\alpha }^{s}(X/Y)=-\sum_{i=1}^{k}\sum_{j=1}^{l}s(x_{i}\cdot y_{j})\cdot \ln\frac{s(x_{i}\cdot y_{j})}{s(y_{j})}.$$

**Proof.**  For every α∈(0,1)∪(1,∞), we have:

$$T_{\alpha }^{s}(X/Y)=\frac{1}{\alpha -1}\left(\sum_{j=1}^{l}s{\left(y_{j}\right)}^{\alpha }-\sum_{i=1}^{k}\sum_{j=1}^{l}s{\left(x_{i}\cdot y_{j}\right)}^{\alpha }\right)=\frac{f(\alpha )}{g(\alpha )},$$

where f and g are continuous functions defined, for every α∈(0,∞), by the equalities:

$$f(\alpha )=\sum_{j=1}^{l}s{\left(y_{j}\right)}^{\alpha }-\sum_{i=1}^{k}\sum_{j=1}^{l}s{\left(x_{i}\cdot y_{j}\right)}^{\alpha },g(\alpha )=\alpha -1.$$

The functions f and g are differentiable and evidently, $\underset{\alpha \to 1}{\lim}g(\alpha )=0.$ Also, it can easily be verified that $\underset{\alpha \to 1}{\lim}f(\alpha )=0.$ Indeed, by Proposition 3, we get:

$$\underset{\alpha \to 1}{\lim}f(\alpha )=\sum_{j=1}^{l}s(y_{j})-\sum_{i=1}^{k}\sum_{j=1}^{l}s(x_{i}\cdot y_{j})=1-\sum_{i=1}^{k}s(x_{i})=1-1=0.$$

Using L’Hôpital’s rule, it follows that $\underset{\alpha \to 1}{\lim}T_{\alpha }^{s}(X/Y)=\underset{\alpha \to 1}{\lim}\frac{f^{\prime }(\alpha )}{g^{\prime }(\alpha )},$ under the assumption that the right hand side exists. It holds $\frac{d}{d\alpha }g(\alpha )=1,$ and:

$$\frac{d}{d\alpha }f(\alpha )=\sum_{j=1}^{l}\frac{d}{d\alpha }\left(s{\left(y_{j}\right)}^{\alpha }\right)-\sum_{i=1}^{k}\sum_{j=1}^{l}\frac{d}{d\alpha }\left(s{\left(x_{i}\cdot y_{j}\right)}^{\alpha }\right)=\sum_{j=1}^{l}s{\left(y_{j}\right)}^{\alpha }\ln s(y_{j})-\sum_{i=1}^{k}\sum_{j=1}^{l}s{\left(x_{i}\cdot y_{j}\right)}^{\alpha }\ln s(x_{i}\cdot y_{j}).$$

It follows that:

$$\begin{array}{l} \underset{\alpha \to 1}{\lim}T_{\alpha }^{s}(X/Y)=\underset{\alpha \to 1}{\lim}f^{\prime }(\alpha )=\sum_{j=1}^{l}s(y_{j})\ln s(y_{j})-\sum_{i=1}^{k}\sum_{j=1}^{l}s(x_{i}\cdot y_{j})\ln s(x_{i}\cdot y_{j})\\ =\sum_{i=1}^{k}\sum_{j=1}^{l}s(x_{i}\cdot y_{j})\cdot \ln s(y_{j})-\sum_{i=1}^{k}\sum_{j=1}^{l}s(x_{i}\cdot y_{j})\cdot \ln s(x_{i}\cdot y_{j})=-\sum_{i=1}^{k}\sum_{j=1}^{l}s(x_{i}\cdot y_{j})\cdot \ln\frac{s(x_{i}\cdot y_{j})}{s(y_{j})}.\;☐ \end{array}$$

**Example** **6.** *Let* $X=(x_{1},x_{2},\ldots ,x_{k})$ *be any partition in a product MV-algebra* (A,⋅), *and* E=(u). *Then:*

$$T_{\alpha }^{s}(X/E)=\frac{1}{\alpha -1}\left(s{\left(u\right)}^{\alpha }-\sum_{i=1}^{k}s{\left(x_{i}\cdot u\right)}^{\alpha }\right)=\frac{1}{\alpha -1}\left(1-\sum_{i=1}^{k}s{\left(x_{i}\right)}^{\alpha }\right)=T_{\alpha }^{s}(X).$$

**Theorem** **7.** *Let* $X=(x_{1},x_{2},\ldots ,x_{k})$ *be any partition in a product MV-algebra* (A,⋅). *Then:*

$$\underset{\alpha \to 1}{\lim}T_{\alpha }^{s}(X)=-\sum_{i=1}^{k}s(x_{i})\cdot \ln s(x_{i}).$$

**Proof.**  The statement is an immediate consequence of the previous theorem; it suffices to put Y=E=(u). ☐

**Theorem** **8.** *For arbitrary partitions* X,Y,Z *in a product MV-algebra* (A,⋅), *it holds:*

*(i)*  $T_{\alpha }^{s}(X/Y)\geq 0;$
*(ii)*  $T_{\alpha }^{s}(X\vee Y/Z)=T_{\alpha }^{s}(X/Z)+T_{\alpha }^{s}(Y/X\vee Z);$
*(iii)*  $T_{\alpha }^{s}(X\vee Y)=T_{\alpha }^{s}(X)+T_{\alpha }^{s}(Y/X).$

**Proof.**  Let $X=(x_{1},x_{2},\ldots ,x_{k}),$ $Y=(y_{1},y_{2},\ldots ,y_{l}),$ $Z=(z_{1},z_{2},\ldots ,z_{m}).$

(i) By Proposition 3, it holds $s(y_{j})=\sum_{i=1}^{k}s(x_{i}\cdot y_{j}),$ for j=1,2,…,l, hence, we can write:
  $$\begin{array}{l} T_{\alpha }^{s}(X/Y)=\frac{1}{\alpha -1}\left(\sum_{j=1}^{l}s{\left(y_{j}\right)}^{\alpha }-\sum_{i=1}^{k}\sum_{j=1}^{l}s{\left(x_{i}\cdot y_{j}\right)}^{\alpha }\right)=\frac{1}{\alpha -1}\left(\sum_{j=1}^{l}s{\left(y_{j}\right)}^{\alpha -1}\sum_{i=1}^{k}s(x_{i}\cdot y_{j})-\sum_{i=1}^{k}\sum_{j=1}^{l}s{\left(x_{i}\cdot y_{j}\right)}^{\alpha }\right)\\ =\frac{1}{\alpha -1}\sum_{i=1}^{k}\sum_{j=1}^{l}s(x_{i}\cdot y_{j})\left(s{\left(y_{j}\right)}^{\alpha -1}-s{\left(x_{i}\cdot y_{j}\right)}^{\alpha -1}\right). \end{array}$$

Suppose that α∈(1,∞). For i=1,2,…,k, j=1,2,…,l, we have $s(x_{i}\cdot y_{j})\leq s(y_{j}),$ which implies that $s{\left(x_{i}\cdot y_{j}\right)}^{\alpha -1}\leq s{\left(y_{j}\right)}^{\alpha -1},$ for i=1,2,…,k, j=1,2,…,l. Since $\frac{1}{\alpha -1}>0,$ for α∈(1,∞), it follows that $T_{\alpha }^{s}(X/Y)\geq 0.$ On the other hand, for α∈(0,1), it holds $s{\left(x_{i}\cdot y_{j}\right)}^{\alpha -1}\geq s{\left(y_{j}\right)}^{\alpha -1},$ for i=1,2,…,k, j=1,2,…,l. In this case $\frac{1}{\alpha -1}<0,$ hence $T_{\alpha }^{s}(X/Y)\geq 0.$

(ii) By direct calculations, we have:
  $$\begin{array}{l} T_{\alpha }^{s}(X/Z)+T_{\alpha }^{s}(Y/X\vee Z)=\frac{1}{\alpha -1}\left(\sum_{k=1}^{m}s{\left(z_{k}\right)}^{\alpha }-\sum_{i=1}^{k}\sum_{k=1}^{m}s{\left(x_{i}\cdot z_{k}\right)}^{\alpha }\right)\\ +\frac{1}{\alpha -1}\left(\sum_{i=1}^{k}\sum_{k=1}^{m}s{\left(x_{i}\cdot z_{k}\right)}^{\alpha }-\sum_{i=1}^{k}\sum_{j=1}^{l}\sum_{k=1}^{m}s{\left(x_{i}\cdot y_{j}\cdot z_{k}\right)}^{\alpha }\right)\\ =\frac{1}{\alpha -1}\left(\sum_{k=1}^{m}s{\left(z_{k}\right)}^{\alpha }-\sum_{i=1}^{k}\sum_{j=1}^{l}\sum_{k=1}^{m}s{\left(x_{i}\cdot y_{j}\cdot z_{k}\right)}^{\alpha }\right)=T_{\alpha }^{s}(X\vee Y/Z). \end{array}$$
(iii) The statement is an immediate consequence of the previous property; it suffices to put Z=E=(u). ☐

By combining the property (iii) from Theorem 8 with Theorem 4, we obtain the following property of conditional Tsallis entropy $T_{\alpha }^{s}(X/Y)$.

**Theorem** **9.** *If partitions* X,Y *in a product MV-algebra* (A,⋅) *are statistically independent with respect to* s, *then:*

$$T_{\alpha }^{s}(X/Y)=T_{\alpha }^{s}(X)+(1-\alpha )\cdot T_{\alpha }^{s}(X)\cdot T_{\alpha }^{s}(Y).$$

**Theorem** **10.** *Let* X,Y *be partitions in a product MV-algebra* (A,⋅). *Then, for* α>1, *it holds:*

$$T_{\alpha }^{s}(X/Y)\leq T_{\alpha }^{s}(X).$$

**Proof.**  Let α>1. Then by the use of the property (iii) from Theorem 8 and Theorem 3, we get:

$$T_{\alpha }^{s}(X/Y)=T_{\alpha }^{s}(X\vee Y)-T_{\alpha }^{s}(Y)\leq T_{\alpha }^{s}(X)+T_{\alpha }^{s}(Y)-T_{\alpha }^{s}(Y)=T_{\alpha }^{s}(X).\;☐$$

To illustrate the result of previous theorem, we provide the following example, which is a continuation of Example 4.

**Example** **7.** *Consider the product MV-algebra* (A,⋅), *the state* s:A→[0,1] *and the partitions* X,Y *from Example 4. We have calculated that* $T_{3}^{s}(X)=0.375,$ $T_{3}^{s}(Y)\dot{=}0.3333,$ $T_{1/2}^{s}(X)\dot{=}0.8284,$ $T_{1/2}^{s}(Y)\dot{=}0.7877.$ *By easy calculations we get that* $T_{3}^{s}(X/Y)\dot{=}0.1146,$ $T_{1/2}^{s}(X/Y)\dot{=}1.0806,$ $T_{3}^{s}(Y/X)=0.0729,$ $T_{1/2}^{s}(Y/X)\dot{=}1.0399.$ *It can be seen that* $T_{3}^{s}(X/Y)<T_{3}^{s}(X),$ *and* $T_{3}^{s}(Y/X)<T_{3}^{s}(Y),$ *which is consistent with the assertion of Theorem 10. On the other hand, we have* $T_{1/2}^{s}(X/Y)>T_{1/2}^{s}(X),$ *and* $T_{1/2}^{s}(Y/X)>T_{1/2}^{s}(Y).$ *The result means that the conditional Tsallis entropy* $T_{\alpha }^{s}(X/Y)$ *of order* α∈(0,1) *does not have the property of monotonicity.*

## 5. The Tsallis Entropy of Dynamical Systems in a Product MV-Algebra

In this section, we introduce and study the concept of the Tsallis entropy of a dynamical system in a product MV-algebra (A,⋅).

**Definition** **8.** ([32]). *By a dynamical system in a product MV-algebra* (A,⋅), *we understand a system* (A,s,τ), *where* s:A→[0,1] *is a state, and* τ:A→A *is a map such that* τ(u)=u, *and, for every* x,y∈A, *the following conditions are satisfied:*

*(i)*  *if*x+y≤u,*then*τ(x)+τ(y)≤u,*and*τ(x+y)=τ(x)+τ(y);
*(ii)*  τ(x⋅y)=τ(x)⋅τ(y);
*(iii)*  s(τ(x))=s(x).

**Remark** **3.** *We say also briefly a product MV-algebra dynamical system instead of a dynamical system in a product MV-algebra.*

**Example** **8.** *Let* (Ω,Σ,μ,T) *be a classical dynamical system. Let us consider the product MV-algebra* (A,⋅) *and the state* s:A→[0,1] *from Example 1. In addition, let us define the mapping* τ:A→A *by the equality* $\tau (I_{E})=I_{E}\circ T=I_{T^{-1}(E)},$ *for every* $I_{E}\in A.$ *Then the system* (A,s,τ) *is a dynamical system in the considered product MV-algebra* (A,⋅).

**Example** **9.** *Let* (Ω,Σ,μ,T) *be a classical dynamical system. Let us consider the product MV-algebra* (A,⋅) *and the state* s:A→[0,1] *from Example 2. If we define the mapping* τ:A→A *by the equality* τ(f)=f∘T, *for every* f∈A, *then it is easy to verify that the system* (A,s,τ) *is a dynamical system in the considered product MV-algebra* (A,⋅).

Let (A,s,τ) be a dynamical system in a product MV-algebra (A,⋅), and $X=(x_{1},x_{2},\ldots ,x_{k})$ be a partition in (A,⋅). Put $\tau (X)=(\tau (x_{1}),\tau (x_{2}),\ldots ,\tau (x_{k})).$ Since $x_{1}+x_{2}+\ldots +x_{k}=u,$ according to Definition 8, we have $\tau (x_{1})+\tau (x_{2})+\ldots +\tau (x_{k})=\tau (x_{1}+x_{2}+\ldots +x_{k})=\tau (u)=u,$ what means that the *k*-tuple τ(X) is a partition in (A,⋅).

**Proposition** **6.** *Let* (A,s,τ) *be a dynamical system in a product MV-algebra* (A,⋅), *and* X,Y *be partitions in* (A,⋅). *Then*

*(i)*  τ(X∨Y)=τ(X)∨τ(Y);
*(ii)*  X≺Y *implies* τ(X)≺τ(Y).

**Proof.**  The property (i) follows from the condition (ii) of Definition 8. Suppose that $X=(x_{1},x_{2},\ldots ,x_{k}),$ $Y=(y_{1},y_{2},\ldots ,y_{l}),$ X≺Y. Then there exists a partition {β(1),β(2),…,β(k)} of the set {1,2,…,l} such that $x_{i}=\sum_{j\in \beta \left(i\right)}y_{j},$ for Therefore, by the condition (i) from Definition 8, we have:

$$\tau (x_{i})=\tau \left(\sum_{j\in \beta \left(i\right)}y_{j}\right)=\sum_{j\in \beta \left(i\right)}\tau (y_{j}),\;\mathrm{for}\;i=1,2,\ldots ,k.$$

However, this means that τ(X)≺τ(Y). ☐

Define $\tau^{2}=\tau \circ \tau ,$ and put $\tau^{n}=\tau \circ \tau^{n-1},$ for n=1,2,…, where $\tau^{0}$ is the identical mapping. It is obvious that the mapping $\tau^{n}:A\to A$ possesses the properties from Definition 8. Hence, for any non-negative integer n, the system $\left(A,s,\tau^{n}\right)$ is a dynamical system in a product MV-algebra (A,⋅).

**Theorem** **11.** *Let* (A,s,τ) *be a dynamical system in a product MV-algebra* (A,⋅), *and* X,Y *be partitions in* (A,⋅). *Then, for any non-negative integer* n, *the following equalities hold:*

*(i)*  $T_{\alpha }^{s}(\tau^{n}(X))=T_{\alpha }^{s}(X);$
*(ii)*  $T_{\alpha }^{s}(\tau^{n}(X)/\tau^{n}(Y))=T_{\alpha }^{s}(X/Y).$

**Proof.**  Suppose that $X=(x_{1},x_{2},\ldots ,x_{k}),$ $Y=(y_{1},y_{2},\ldots ,y_{l}).$

(i) Since for any non-negative integer n, and i=1,2,…,k, it holds $s(\tau^{n}(x_{i}))=s(x_{i}),$ we obtain:
  $$T_{\alpha }^{s}(\tau^{n}(X))=\sum_{i=1}^{k}l_{\alpha }\left(s(\tau^{n}(x_{i}))\right)=\sum_{i=1}^{k}l_{\alpha }\left(s(x_{i})\right)=T_{\alpha }^{s}(X).$$
(ii) Based on the same argument, we get:
  $$\begin{array}{l} T_{\alpha }^{s}(\tau^{n}(X)/\tau^{n}(Y))=\frac{1}{\alpha -1}\left(\sum_{j=1}^{l}s{\left(\tau^{n}(y_{j})\right)}^{\alpha }-\sum_{i=1}^{k}\sum_{j=1}^{l}s{\left(\tau^{n}(x_{i}\cdot y_{j})\right)}^{\alpha }\right)\\ =\frac{1}{\alpha -1}\left(\sum_{j=1}^{l}s{\left(y_{j}\right)}^{\alpha }-\sum_{i=1}^{k}\sum_{j=1}^{l}s{\left(x_{i}\cdot y_{j}\right)}^{\alpha }\right)=T_{\alpha }^{s}(X/Y).\;☐ \end{array}$$

**Theorem** **12.** *Let* (A,s,τ) *be a dynamical system in a product MV-algebra* (A,⋅), *and* X *be a partition in* (A,⋅). *Then, for* n=2,3,…, *the following equality holds:*

$$T_{\alpha }^{s}(\vee_{k=0}^{n-1}\tau^{k}(X))=T_{\alpha }^{s}(X)+\sum_{i=1}^{n-1}T_{\alpha }^{s}(X/\vee_{k=1}^{i}\tau^{k}(X)).$$

**Proof.**  We use proof by mathematical induction on n, starting with n=2. For n=2, the claim holds by the property (iii) of Theorem 8. We suppose that the claim holds for a given integer n>1, and we will prove that it holds for n+1. By the property (i) of Theorem 11, we get:

$$T_{\alpha }^{s}(\vee_{k=1}^{n}\tau^{k}(X))=T_{\alpha }^{s}(\tau (\vee_{k=0}^{n-1}\tau^{k}(X)))=T_{\alpha }^{s}(\vee_{k=0}^{n-1}\tau^{k}(X)).$$

Therefore, using the property (iii) of Theorem 8 and our inductive hypothesis, we obtain:

$$\begin{array}{l} T_{\alpha }^{s}(\vee_{k=0}^{n}\tau^{k}(X))=T_{\alpha }^{s}((\vee_{k=1}^{n}\tau^{k}(X))\vee X)=T_{\alpha }^{s}(\vee_{k=1}^{n}\tau^{k}(X))+T_{\alpha }^{s}(X/\vee_{k=1}^{n}\tau^{k}(X))\\ =T_{\alpha }^{s}(\vee_{k=0}^{n-1}\tau^{k}(X))+T_{\alpha }^{s}(X/\vee_{k=1}^{n}\tau^{k}(X))\\ =T_{\alpha }^{s}(X)+\sum_{i=1}^{n-1}T_{\alpha }^{s}(X/\vee_{k=1}^{i}\tau^{k}(X))+T_{\alpha }^{s}(X/\vee_{k=1}^{n}\tau^{k}(X))\\ =T_{\alpha }^{s}(X)+\sum_{i=1}^{n}T_{\alpha }^{s}(X/\vee_{k=1}^{i}\tau^{k}(X)). \end{array}$$

In conclusion, the claim is obtained by the principle of mathematical induction. ☐

In the following, we will define the Tsallis entropy of a dynamical system (A,s,τ). First, we define the Tsallis entropy of τ relative to a partition X in (A,⋅). Then we remove the dependence on X to get the Tsallis entropy of a dynamical system (A,s,τ). The following proposition will be needed.

**Proposition** **7.** *Let* (A,s,τ) *be a dynamical system in a product MV-algebra* (A,⋅), *and* X *be a partition in* (A,⋅). *Then, for* α>1, *there exists the following limit:*

$$\underset{n\to \infty }{\lim}\frac{1}{n}T_{{}^{\alpha }}^{s}(\vee_{k=0}^{n-1}\tau^{k}(X)).$$

**Proof.**  Put $c_{n}=T_{{}^{\alpha }}^{s}(\vee_{k=0}^{n-1}\tau^{k}(X)),$ for n=1,2,…. Then the sequence ${\left\{c_{n}\right\}}_{n=1}^{\infty }$ is a sequence of non-negative real numbers with the property $c_{r+s}\leq c_{r}+c_{s},$ for every natural numbers r,s. Indeed, by means of sub-additivity of Tsallis entropy $T_{\alpha }^{s}(X)$ for α>1, and the property (i) from Theorem 11, we have:

$$\begin{array}{l} c_{r+s}=T_{{}^{\alpha }}^{s}(\vee_{k=0}^{r+s-1}\tau^{k}(X))\leq T_{{}^{\alpha }}^{s}(\vee_{k=0}^{r-1}\tau^{k}(X))+T_{{}^{\alpha }}^{s}(\vee_{k=r}^{r+s-1}\tau^{k}(X))\\ =c_{r}+T_{{}^{\alpha }}^{s}(\tau^{r}(\vee_{k=0}^{s-1}\tau^{k}(X)))=c_{r}+T_{{}^{\alpha }}^{s}(\vee_{k=0}^{s-1}\tau^{k}(X))=c_{r}+c_{s}. \end{array}$$

The result guarantees (in view of Theorem 4.9, [55]) the existence of $\underset{n\to \infty }{\lim}\frac{1}{n}c_{n}.$ ☐

**Definition** **9.** *Let*(A,s,τ)*be a dynamical system in a product MV-algebra*(A,⋅),*and*X*be a partition in*(A,⋅).*Then we define, for*α>1,*the Tsallis entropy of*τ*relative to*X by:

$$T_{{}^{\alpha }}^{s}(\tau ,X)=\underset{n\to \infty }{\lim}\frac{1}{n}T_{{}^{\alpha }}^{s}(\vee_{k=0}^{n-1}\tau^{k}(X)).$$

**Remark** **4.** *Consider any dynamical system* (A,s,τ) *in a product MV-algebra* (A,⋅), *and the partition* E=(u). *Evidently,* $\vee_{k=0}^{n-1}\tau^{k}(E)=E,$ *and* $T_{{}^{\alpha }}^{s}(\tau ,E)=\underset{n\to \infty }{\lim}\frac{1}{n}T_{{}^{\alpha }}^{s}(\vee_{k=0}^{n-1}\tau^{k}(E))=\underset{n\to \infty }{\lim}\frac{1}{n}T_{{}^{\alpha }}^{s}(E)=0.$

**Theorem** **13.** *Let* (A,s,τ) *be a dynamical system in a product MV-algebra* (A,⋅), *and* X *be a partition in* (A,⋅). *Then, for* α>1, *and for any non-negative integer* r, *the following equality holds:*

$$T_{{}^{\alpha }}^{s}(\tau ,X)=T_{{}^{\alpha }}^{s}(\tau ,\vee_{i=0}^{r}\tau^{i}(X)).$$

**Proof.**  Using Definition 9, we can write:

$$\begin{array}{l} T_{{}^{\alpha }}^{s}(\tau ,\vee_{i=0}^{r}\tau^{i}(X))=\underset{n\to \infty }{\lim}\frac{1}{n}T_{{}^{\alpha }}^{s}(\vee_{k=0}^{n-1}\tau^{k}(\vee_{i=0}^{r}\tau^{i}(X)))\\ =\underset{n\to \infty }{\lim}\frac{r+n}{n}\cdot \frac{1}{r+n}T_{{}^{\alpha }}^{s}(\vee_{k=0}^{r+n-1}\tau^{k}(X))\\ =\underset{n\to \infty }{\lim}\frac{1}{r+n}T_{{}^{\alpha }}^{s}(\vee_{k=0}^{r+n-1}\tau^{k}(X))=T_{{}^{\alpha }}^{s}(\tau ,X).\;☐ \end{array}$$

**Theorem** **14.** *Let* (A,s,τ) *be a dynamical system in a product MV-algebra* (A,⋅), *and* X,Y *be partitions in* (A,⋅) *such that* X≺Y. *Then, for* α>1, *it holds* $T_{\alpha }^{s}(\tau ,X)\leq T_{\alpha }^{s}(\tau ,Y).$

**Proof.**  Let X≺Y. By Propositions 2 and 6, we have $\vee_{k=0}^{n-1}\tau^{k}(X)≺\vee_{k=0}^{n-1}\tau^{k}(Y),$ for n=1,2,…. Therefore, by Theorem 2, we get:

$$T_{\alpha }^{s}(\vee_{k=0}^{n-1}\tau^{k}(X))\leq T_{\alpha }^{s}(\vee_{k=0}^{n-1}\tau^{k}(Y)).$$

Consequently, dividing by n and letting n→∞, we get $T_{\alpha }^{s}(\tau ,X)\leq T_{\alpha }^{s}(\tau ,Y).$ ☐

**Definition** **10.** *The Tsallis entropy of a dynamical system* (A,s,τ) *in a product MV-algebra* (A,⋅) *is defined, for* α>1, *by:*

$$T_{{}^{\alpha }}^{s}(\tau )=\sup\{T_{{}^{\alpha }}^{s}(\tau ,X);X\;\mathrm{is}\;a\;\mathrm{partition}\;\mathrm{in}\;\left(A,\cdot \right)\}.$$

**Theorem** **15.** *Let* (A,s,τ) *be a dynamical system in a product MV-algebra* (A,⋅). *Then, for* α>1, *and every natural number* k, *it holds* $T_{{}^{\alpha }}^{s}(\tau^{k})=k\cdot T_{{}^{\alpha }}^{s}(\tau ).$

**Proof.**  Let X be a partition in (A,⋅). Then, for every natural number k, we have:

$$\begin{array}{l} T_{{}^{\alpha }}^{s}(\tau^{k},\vee_{j=0}^{k-1}\tau^{j}(X))=\underset{n\to \infty }{\lim}\frac{1}{n}T_{{}^{\alpha }}^{s}(\vee_{i=0}^{n-1}{\left(\tau^{k}\right)}^{i}(\vee_{j=0}^{k-1}\tau^{j}(X))\\ =\underset{n\to \infty }{\lim}\frac{1}{n}T_{{}^{\alpha }}^{s}(\vee_{i=0}^{n-1}\vee_{j=0}^{k-1}\tau^{k}{}^{i+j}(X))=\underset{n\to \infty }{\lim}\frac{nk}{n}\frac{1}{nk}T_{{}^{\alpha }}^{s}(\vee_{j=0}^{nk-1}\tau^{j}(X))=k\cdot T_{{}^{\alpha }}^{s}(\tau ,X). \end{array}$$

Hence, we obtain:

$$k\cdot T_{{}^{\alpha }}^{s}(\tau )=k\cdot \sup\{T_{{}^{\alpha }}^{s}(\tau ,X);X\;\mathrm{is}\;a\;\mathrm{partition}\;\mathrm{in}\;\left(A,\cdot \right)\}\;=\sup\{T_{{}^{\alpha }}^{s}(\tau^{k},\vee_{j=0}^{k-1}\tau^{j}(X));X\;\mathrm{is}\;a\;\mathrm{partition}\;\mathrm{in}\;\left(A,\cdot \right)\}\;\leq \sup\{T_{{}^{\alpha }}^{s}(\tau^{k},Y);Y\;\mathrm{is}\;a\;\mathrm{partition}\;\mathrm{in}\;\left(A,\cdot \right)\}=T_{{}^{\alpha }}^{s}(\tau^{k}).$$

On the other hand, by Proposition 1, we have $X≺\vee_{j=0}^{k-1}\tau^{j}(X).$ Hence, by Theorem 14, we obtain:

$$T_{{}^{\alpha }}^{s}(\tau^{k},X)\leq T_{{}^{\alpha }}^{s}(\tau^{k},\vee_{j=0}^{k-1}\tau^{j}(X))=k\cdot T_{{}^{\alpha }}^{s}(\tau ,X).$$

This implies that:

$$T_{{}^{\alpha }}^{s}(\tau^{k})=\sup\{T_{{}^{\alpha }}^{s}(\tau^{k},X);X\;\mathrm{is}\;a\;\mathrm{partition}\;\mathrm{in}\;\left(A,\cdot \right)\}\;\leq k\cdot \sup\{T_{{}^{\alpha }}^{s}(\tau ,X);X\;\mathrm{is}\;a\;\mathrm{partition}\;\mathrm{in}\;\left(A,\cdot \right)\}=k\cdot T_{{}^{\alpha }}^{s}(\tau ).\;☐$$

**Definition** **11.** *Two product MV-algebra dynamical systems* $(A_{1},s_{1},\tau_{1}),$ $\left(A_{2},s_{2},\tau_{2}\right)$ *are called isomorphic, if there exists some one-to-one and onto map* $\Phi :A_{1}\to A_{2}$ *such that* $\Phi (u_{1})=u_{2},$ *and, for every* $x,y\in A_{1},$ *the following conditions are satisfied:*

*(i)*  Φ(x⋅y)=Φ(x)⋅Φ(y);
*(ii)*  *if* $x+y\leq u_{1},$ *then* Φ(x+y)=Φ(x)+Φ(y);
*(iii)*  $s_{2}(\Phi (x))=s_{1}(x);$
*(iv)*  $\Phi (\tau_{1}(x))=\tau_{2}(\Phi (x)).$

*In this case,* Φ *is said to be an isomorphism.*

**Proposition** **8.** *Let* $(A_{1},s_{1},\tau_{1}),$ $\left(A_{2},s_{2},\tau_{2}\right)$ *be isomorphic product MV-algebra dynamical systems, and* $\Phi :A_{1}\to A_{2}$ *be an isomorphism between them. Then, for the inverse* $\Phi^{-1}:A_{2}\to A_{1},$ *the following properties are satisfied:*

*(i)*  $\Phi^{-1}(x\cdot y)=\Phi^{-1}(x)\cdot \Phi^{-1}(y),$ *for every* $x,y\in A_{2};$
*(ii)*  *if* $x,y\in A_{2}$ *such that* $x+y\leq u_{2},$ *then* $\Phi^{-1}(x+y)=\Phi^{-1}(x)+\Phi^{-1}(y);$
*(iii)*  $s_{1}(\Phi^{-1}(x))=s_{2}(x),$ *for every* $x\in A_{2};$
*(iv)*  $\Phi^{-1}(\tau_{2}(x))=\tau_{1}(\Phi^{-1}(x)),$ *for every* $x\in A_{2}.$

**Proof.**  The map $\Phi :A_{1}\to A_{2}$ is bijective, therefore, for every $x,y\in A_{2},$ there exist $x^{\prime },y^{\prime }\in A_{1}$ such that $\Phi^{-1}(x)=x^{\prime },$ and $\Phi^{-1}(y)=y^{\prime }.$

(i) Let $x,y\in A_{2}.$ Then we get:
  $$\Phi^{-1}(x\cdot y)=\Phi^{-1}(\Phi (x^{\prime })\cdot \Phi (y^{\prime }))=\Phi^{-1}(\Phi (x^{\prime }\cdot y^{\prime }))=x^{\prime }\cdot y^{\prime }=\Phi^{-1}(x)\cdot \Phi^{-1}(y).$$
(ii) Let $x,y\in A_{2}$ such that $x+y\leq u_{2}.$ Then $x^{\prime }+y^{\prime }\leq u_{1},$ and, therefore, we have:
  $$\Phi^{-1}(x+y)=\Phi^{-1}(\Phi (x^{\prime })+\Phi (y^{\prime }))=\Phi^{-1}(\Phi (x^{\prime }+y^{\prime }))=x^{\prime }+y^{\prime }=\Phi^{-1}(x)+\Phi^{-1}(y).$$
(iii) Let $x\in A_{2}.$ Then $s_{2}(x)=s_{2}(\Phi (x^{\prime }))=s_{1}(x^{\prime })=s_{1}(\Phi^{-1}(x)).$
(iv) Let $x\in A_{2}.$ Then $\Phi^{-1}(\tau_{2}(x))=\Phi^{-1}(\tau_{2}(\Phi (x^{\prime })))=\Phi^{-1}(\Phi (\tau_{1}(x^{\prime })))=\tau_{1}(x^{\prime })=\tau_{1}(\Phi^{-1}(x)).\;☐$

**Theorem** **16.** *Let* $(A_{1},s_{1},\tau_{1}),$ $\left(A_{2},s_{2},\tau_{2}\right)$ *be isomorphic product MV-algebra dynamical systems, and* α>1. *Then:*

$$T_{{}^{\alpha }}^{s_{1}}(\tau_{1})=T_{{}^{\alpha }}^{s_{2}}(\tau_{2}).$$

**Proof.**  Let $\Phi :A_{1}\to A_{2}$ be an isomorphism between dynamical systems $(A_{1},s_{1},\tau_{1}),$ $(A_{2},s_{2},\tau_{2}).$ Consider a partition $X=(x_{1},x_{2},\ldots ,x_{k})$ in a product MV-algebra $(A_{1},\cdot ).$ Then $x_{1}+x_{2}+\ldots +x_{k}=u_{1},$ and therefore, by the condition (ii) of Definition 11, it holds $\Phi (x_{1})+\Phi (x_{2})+\ldots +\Phi (x_{k})$ $=\Phi (x_{1}+x_{2}+\ldots +x_{k})=\Phi (u_{1})=u_{2}.$ This means that the *k*-tuple $\Phi (X)=(\Phi (x_{1}),\Phi (x_{2}),\ldots ,\Phi (x_{k}))$ is a partition in a product MV-algebra $(A_{2},\cdot ).$ Moreover, according to the condition (iii) of Definition 11, we have:

$$T_{{}^{\alpha }}^{s_{2}}(\Phi (X))=\sum_{i=1}^{k}l_{\alpha }(s_{2}(\Phi (x_{i})))=\sum_{i=1}^{k}l_{\alpha }(s_{1}(x_{i}))=T_{\alpha }^{s_{1}}(X).$$

Hence, using the conditions (iv), and (i) of Definition 11, we get:

$$\begin{array}{l} T_{{}^{\alpha }}^{s_{2}}(\vee_{k=0}^{n-1}\tau_{2}^{k}(\Phi (X)))=T_{{}^{\alpha }}^{s_{2}}(\vee_{k=0}^{n-1}\Phi (\tau_{1}^{k}(X)))\\ =T_{{}^{\alpha }}^{s_{2}}(\Phi (\vee_{k=0}^{n-1}\tau_{1}^{k}(X)))=T_{\alpha }^{s_{1}}(\vee_{k=0}^{n-1}\tau_{1}^{k}(X)). \end{array}$$

Therefore, dividing by n and letting n→∞, we obtain:

$$T_{{}^{\alpha }}^{s_{2}}(\tau_{2},\Phi (X))=\underset{n\to \infty }{\lim}\frac{1}{n}T_{{}^{\alpha }}^{s_{2}}(\vee_{k=0}^{n-1}\tau_{2}^{k}(\Phi (X)))\underset{n\to \infty }{=\lim}\frac{1}{n}T_{\alpha }^{s_{1}}(\vee_{k=0}^{n-1}\tau_{1}^{k}(X))=T_{\alpha }^{s_{1}}(\tau_{1},X).$$

This implies that:

$$\{T_{\alpha }^{s_{1}}(\tau_{1},X);X\;\mathrm{is}\;a\;\mathrm{partition}\;\mathrm{in}\;\left(A_{1},\cdot \right)\}\subset \{T_{{}^{\alpha }}^{s_{2}}(\tau_{2},Y);Y\;\mathrm{is}\;a\;\mathrm{partition}\;\mathrm{in}\;\left(A_{2},\cdot \right)\},$$

and consequently:

$$T_{{}^{\alpha }}^{s_{1}}(\tau_{1})=\sup\{T_{{}^{\alpha }}^{s_{1}}(\tau_{1},X);X\;\mathrm{is}\;a\;\mathrm{partition}\;\mathrm{in}\;\left(A_{1},\cdot \right)\}\;\leq \sup\{T_{{}^{\alpha }}^{s_{2}}(\tau_{2},Y);Y\;\mathrm{is}\;a\;\mathrm{partition}\;\mathrm{in}\;\left(A_{2},\cdot \right)\}=T_{{}^{\alpha }}^{s_{2}}(\tau_{2}).$$

The converse $T_{{}^{\alpha }}^{s_{2}}(\tau_{2})\leq T_{{}^{\alpha }}^{s_{1}}(\tau_{1})$ can be obtained in a similar way; according to Proposition 8, it suffices to consider the inverse $\Phi^{-1}:A_{2}\to A_{1}.$ ☐

**Remark** **5.** *It trivially follows from Theorem 16 that if* $T_{{}^{\alpha }}^{s_{1}}(\tau_{1})\neq T_{{}^{\alpha }}^{s_{2}}(\tau_{2}),$ *then the corresponding dynamical systems* $(A_{1},s_{1},\tau_{1}),$ $\left(A_{2},s_{2},\tau_{2}\right)$ *are not isomorphic. This means that some product MV-algebra dynamical systems can be distinguished due to their different Tsallis entropies.*

## 6. Conclusions

In this article we dealt with the mathematical modelling of Tsallis entropy in product MV-algebras. Our results are given in Section 3, Section 4 and Section 5. In Section 3 we have introduced the notion of Tsallis entropy $T_{\alpha }^{s}(X)$ of a partition *X* in a product MV-algebra (A,⋅), and we examined properties of this entropy measure. In Section 4 we have defined and studied the conditional Tsallis entropy of partitions in this algebraic structure. It has been shown that the proposed concepts are consistent, in the case of the limit of α→1, with the Shannon entropy expressed in nats, defined and studied in Reference [31]. Moreover, putting α=2 in the proposed definitions, we obtain the logical entropy of partitions in a product MV-algebra defined and studied in Reference [8].

Section 5 was devoted to the mathematical modelling of Tsallis entropy in product MV-algebra dynamical systems. From Example 8 it follows that the notion of product MV-algebra dynamical system is a generalization of the concept of classical dynamical system. We have shown that the Tsallis entropy is invariant under isomorphism of product MV-algebra dynamical systems.

In the proofs we used L’Hôpital’s rule and the known Jensen inequality. To illustrate the results, we have provided several numerical examples. In Example 2, we have mentioned that the full tribe of fuzzy sets is a special case of product MV-algebras; hence, all the results of this article can be directly applied to this family of fuzzy sets. We remind that a fuzzy subset of a non-empty set Ω is any mapping f:Ω→[0,1], where the value f(ω) is interpreted as the degree of belonging of element ω of Ω to the fuzzy set f (cf. [16]). In Reference [56], Atanassov has generalized the Zadeh fuzzy set theory by introducing the idea of an intuitionistic fuzzy set (IF-set), a set that has the degree of belonging as well as the degree of non-belonging with each of its elements. From the point of view of application, it should be noted that for a given class F of IF-sets can be created an MV-algebra A such that F can be inserted to A. Also the operation of product on F can be defined by such a way that the corresponding MV-algebra is a product MV-algebra. Therefore, the presented results are also applicable to the case of IF-sets.
